# The evolution of multi-gene families and metabolic pathways in the evening primroses (*Oenothera*: Onagraceae): A comparative transcriptomics approach

**DOI:** 10.1371/journal.pone.0269307

**Published:** 2022-06-24

**Authors:** Eunice Kariñho-Betancourt, David Carlson, Jessie Hollister, Axel Fischer, Stephan Greiner, Marc T. J. Johnson

**Affiliations:** 1 Department of Biology, University of Toronto Mississauga, Mississauga, Ontario, Canada; 2 Department of Ecology and Evolution, Stony Brook University, Stony Brook, New York, United States of America; 3 Max Planck Institute of Molecular Plant Physiology, Potsdam-Golm, Germany; Imperial College London, UNITED KINGDOM

## Abstract

The plant genus *Oenothera* has played an important role in the study of plant evolution of genomes and plant defense and reproduction. Here, we build on the 1kp transcriptomic dataset by creating 44 new transcriptomes and analyzing a total of 63 transcriptomes to present a large-scale comparative study across 29 *Oenothera* species. Our dataset included 30.4 million reads per individual and 2.3 million transcripts on average. We used this transcriptome resource to examine genome-wide evolutionary patterns and functional diversification by searching for orthologous genes and performed gene family evolution analysis. We found wide heterogeneity in gene family evolution across the genus, with section *Oenothera* exhibiting the most pronounced evolutionary changes. Overall, more significant gene family expansions occurred than contractions. We also analyzed the molecular evolution of phenolic metabolism by retrieving proteins annotated for phenolic enzymatic complexes. We identified 1,568 phenolic genes arranged into 83 multigene families that varied widely across the genus. All taxa experienced rapid phenolic evolution (fast rate of genomic turnover) involving 33 gene families, which exhibited large expansions, gaining about 2-fold more genes than they lost. Upstream enzymes phenylalanine ammonia-lyase (PAL) and 4-coumaroyl: CoA ligase (4CL) accounted for most of the significant expansions and contractions. Our results suggest that adaptive and neutral evolutionary processes have contributed to *Oenothera* diversification and rapid gene family evolution.

## Introduction

The evening primrose plant genus *Oenothera* (Onagraceae), has served as a model for addressing numerous problems in plant biology during the past 120 years [1]. Pioneering studies in the late 19th and early 20th centuries using *O*. *lamarckiana* (= *O*. *glazioviana*) and other taxa, contributed to the rediscovery of Mendel’s laws of inheritance [2], the formulation of the mutation theory [3–6], helped prove the chromosomal theory of inheritance [7,8], and provided among the earliest examples of genetic self-incompatibility in plant mating [9,10]. The combination of cytogenetics and genetics, first applied to *Oenothera* at the level of the population, contributed to elucidating the role of chromosomal translocations in genome rearrangements, meiotic behavior, and recombination, as well as the role of translocations and speciation [11–14]. In addition, evening primroses are a model system for the study of the genetics, genomics and evolution of plastids in which, for example, the role of the plastids for plant speciation was recognized [15–17], the contribution of the cytoplasmic genetic elements to plant morphology was discovered [18,19], and the theory of selfish cytoplasmic elements developed [20]. In molecular population genetics, the first use of allozyme markers in plants was performed in *Oenothera biennis* [21,22], in which it was shown that populations contained unexpectedly high levels of genetic variation. Finally, *Oenothera* and the Onagraceae at large are among the best studied groups in plant taxonomy and systematics [10,23–34].

Although *Oenothera* has played a fundamental role in plant biology for over a century [1,13], the development of modern genetic and genomic tools has been relatively slow compared to other model systems. Early genomic resources included a complete plastid genome sequence of *O*. *elata* [35], which was expanded to include all five plastome types in *Oenothera* subsection *Oenothera*, allowing for comparative analysis of chloroplast evolution [36,37]. PCR-based dominant amplified fragment length polymorphisms (AFLPs), codominant simple-sequence repeats (SSR), cleaved amplified polymorphic sequence (CAPS), and single sequence length polymorphism (SSLP) marker systems for both the nuclear genomes and plastomes were developed over a decade ago [38,39], which allowed for the creation of a genetic linkage map in *Oenothera* [40]. Progress in molecular biology and sequencing technology allowed for the creation of early EST libraries [41]. In parallel, cytological techniques have continued to advance, including the development of fluorescence *in situ* hybridization [42,43].

The development of these molecular resources has allowed for recent advances in genetics, evolutionary biology, and systematics, and opened new interdisciplinary directions. For example, these resources allowed the first test in plants of multiple predictions stemming from theory of the evolution of sex [40,44–51]. Recent research also led to important advances in the study of plant defense evolution [52–59], and eco-evolutionary dynamics within communities [60–68]. Further, genomic tools have made it possible to determine the role of nuclear-organellar incompatibilities in speciation [69,70], mechanisms of mutations in plastid genome evolution [71], novel mechanisms in chloroplast gene expression [72], and the identification of chloroplast genes involved in plastid transmission [73]. The advances in cytological methods have further improved our understanding of meiosis, and how translocations in *Oenothera* lead to the formation of chromosomal rings in diakinesis [42,43]. Additionally, metabolomic methods [74] have been combined with phylogenetic and genetic approaches to disentangle the role of secondary compounds in plant defense evolution over micro- and macroevolutionary timescales [53,56,59,75–77]. Finally, molecular research in *Oenothera* has made it possible to refine our understanding of *Oenothera* systematics [33,34,44,45,50,78,79], which resulted in key revisions to the taxonomy of the Onagraceae [25].

Recently, as part of the One Thousand Plant Transcriptomes Initiative (1kp project), more than 1000 species were sequenced, including 18 *Oenothera* species, to provide a robust phylogenomic framework for examining the evolution of green plants [80–82]. Here, we combined the 1kp *Oenothera* dataset with 44 transcriptomes separately generated for an additional 11 species (see [49]) and reassembled 19 transcriptomes to greatly increase the number and quality of the *Oenothera* transcriptome resource. We develop comparative transcriptomic tools across the genus and show their utility in understanding the genomic and functional diversification.

*Oenothera* is a monophyletic genus within the Onagraceae family, which is nested within the Myrtales order and the Rosids subclass. The base number of chromosomes is X = N = 7 and evening primrose are usually diploid, but a few species (not studied here) contain polyploid lineages as high as N = 28 [83]. The genus is comprised of 151 species of perennial and annual herbs that are native to North and South America [25]. These species occur throughout boreal, temperate, montane, arid and coastal habitats. *Oenothera* is well known for having variable reproductive and genetic systems. Many species are outcrossing, which often involves genetic self-incompatibility as well as herkogamous flowers that are mainly pollinated by moths and specialized bees [84–86]. Some species are self-pollinating [87], whereas ca. 45 species have a specialized genetic system known as permanent translocation heterozygosity (PTH) [88], which results in plants being functionally asexual because of a loss of recombination and segregation, and a balanced lethal genetic system whereby only heterogametic haploid chromosome sets can form viable offspring [1,13].

*Oenothera* has served as a model for the study of plant secondary metabolism, particularly as it relates to phenolic metabolism. All members of the genus produce phenolic compounds, including flavonoids and hydrolysable tannins [44,56,59]. Phenolic compounds derived from the amino acid L-phenylalanine via deamination by L-phenylalanine ammonia lyase (PAL). Several classes of phenolics follow the shikimic acid pathway in combination with the mevalonate-acetate route. Different biosynthetic paths of phenolics can be organized into a ‘core’ phenylpropanoid pathway, including upstream enzymes: PAL, the member of P450s family cinnamate 4-hydroxylase (C4H) and 4-coumarate coenzyme A ligase (4CL), which feeding further specific pathways [89,90]. Complex phenylpropanoids like flavonoids, flavonols and tannins involve specific mid- and downstream enzymes such as chalcone synthase (CHS) and chalcone isomerase (CHI) to yield their true backbone, and flavonoid 3´-hydroxylase (F3´H), flavanone 3-hydroxylase (F3H) and flavonol synthase (FLS) (S1 Fig), which catalyze terminal biosynthetic branches [91,92]. Phenolics are well known stress response metabolites, with induction of phenylpropanoid metabolism and accumulation of phenolic compounds especially observed in response to biotic stresses such as herbivory [93–95]. Evening primrose is consumed by many insect herbivores [46,61], and plants defend themselves with a diverse arsenal of phenolics. Several studies have shown that specific phenolic compounds reduce herbivore performance [77], and herbivores impose selection that drives rapid evolution of these metabolites [52,53]. Investment in secondary metabolites varies biogeographically both within [55] and between species [59]. Moreover, a loss of sex through PTH is associated with reduced levels of defense against generalist insects in *Oenothera* [52].

We used vegetative transcriptomes for a broad taxonomic sampling of *Oenothera* species and subspecies to characterize gene family evolution and gene expression. After providing a thorough transcriptomic overview, we ask the following questions. How have gene families diverged among species and/or clades? How can this transcriptomic analysis be used to examine the evolution of secondary metabolism and defenses in PTH and sexual plants? Answering these questions will contribute to understanding the molecular and phenotypic evolution of this important system.

## Materials and methods

### Species sampling

The *Oenothera* genus is arranged into two major clades (A and B), which include 18 sections and 18 subsections [25]. We sampled 29 species from seven subclades including diverse sections and subsections of the genus *Oenothera*. The sampling also comprises five subspecies from two species, for a total of 32 taxa. This included sampling 13 sexual species and 16 functionally asexual PTH species across clades. Species’ naming authority, source of seeds, and voucher information associated with the 1kp project and additional transcriptome sampling at the NC State Genomic Facility are provided in S1 Table. We also sampled 3–5 populations from each of 11 species to give insight into genetic variation within species, but are not described here since the focus of this paper was on the comparative context of the transcriptomic analysis. These species provide an excellent platform for addressing evolutionary questions since they maximize diversity across the genus *Oenothera*.

### Plant materials

Seeds of 29 *Oenothera* species (32 taxa including subspecies) were started in Petri dishes. All species except for the *Gaura* section (i.e., *O*. *filiformis*, *O*. *gaura*, and *O*. *suffulta*) were germinated in 55 mm Petri dishes on filter paper soaked in 1mL of 0.1% agarose solution in filtered water, at 4 ºC for 18–24 hours and then left in a windowsill that received full sun for 3–4 hours per day. All *Gaura* fruits were soaked in water as described above but the entire indehiscent capsules were planted 1 cm below moist soil and placed under fluorescent lights for germination. Seeds were transplanted to soil when roots and cotyledons emerged from seeds. All plants were planted in 150 mL pots, in sterilized Ready Earth Seedling and Plug Soil Mixture (Sungro Horticulture, Canada) soil and watered *ad libitum* with DI water with 1/3 strength Hoaglands solution. Plants were grown under 16:8, L:D cycle with 25:20°C (L:D) with fluorescent and incandescent lights. Plants were harvested when the 4^th^ true leaf was 1/4 the area of 3^rd^ true leaf, for all plants. Leaves were flash frozen in liquid N_2_ and placed in a -80 ºC freezer. For further details on *Oenothera* culture and propagation (see [96]).

### Sequencing and transcriptome assembly

Total RNA was isolated from leaf tissue of 63 individuals from *Oenothera* spp. following a CTAB/Acid Phenol/Silica Membrane method [protocol 9; 49,80]. This total RNA was then purified into mRNA using the dyna bead mRNA purification kit [80], and employed to prepared 63 TrueSeq libraries for sequencing. Libraries from 50 samples were sequenced under a pair-end mode using Illumina HiSeq as part of the One Thousand Plant Transcriptomes Initiative (1kp project) [80,81], and an additional 13 samples were sequenced under a single-end mode (13 samples) using GAIIx (S1 Table) at the NC State Genomic Sciences Laboratory (https://research.ncsu.edu/gsl/). All RNA-seq reads (SRA project number: PRJEB4922) were newly trimmed and filtered for quality using fastp v.0.20.0 as part of the current paper [97]. The “—cut_tail” flag was used to truncate reads if a 4 bp window fell below an average PHRED quality score of 20. Quality-controlled RNA-seq reads were then assembled into transcripts using Trinity v2.11.0 [98,99] using default settings except for CPU and memory allocation. Assembly metrics were generated using Trinity’s ‘TrinityStats.pl’.

### Functional annotation

Each assembly was functionally annotated using the Trinotate v3.2.1 pipeline [100]. Briefly, we used Transdecoder v5.5.0 (https://github.com/TransDecoder/TransDecoder) to find putative protein-coding sequences (CDS) in each transcriptome. Each set of CDS and translated amino acid sequences were then searched using blastp v2.5.0 with a maximum e-value of 1e-5 [101] and HMMER v3.3’s hmmscan with default settings against the Swiss-Prot (last accessed July 13, 2020; [102]) and Pfam (last accessed July 13, 2020; [103]) databases. As part of the Trinotate pipeline, we also used SignalP v4.1 [104] to predict signal peptides, TMHMM v2.0c [105] to find transmembrane helices, and RNAmmer v1.2 [106] to predict RNA genes. These four programs were run using default settings, after which the annotation results were loaded into an SQLite database and summarized with Trinotate’s “report” option. To summarize the functional annotation results for each taxon, we counted the number of annotations across the three main GO categories—cellular component, molecular function, and biological process—assigned by Trinotate based on blastp matches between each predicted protein and sequences in the Swiss-Prot database. In addition, we used GOATOOLS v.0.8.2 [107] to further summarize the orthogroup functional annotation results into a set of discrete categories of particular interest. The completeness of the transcriptome gene space was evaluated by using Benchmarking Universal Single-Copy Orthologs (BUSCO), and PLAZA comparative resources. Both BUSCO and PLAZA coreGFs currently define different sets of conserved genes to model the expected gene space at different evolutionary scales within diverse lineages. We performed the BUSCO v.3.0.2 analysis with the transcriptome mode option and the lineage set to embryophyta_odb9 [108], whereas the PLAZA v.4.0 ‘green plants’ coreGFs was set to Rosids [109].

### Ortholog identification and phylogenetic inference

A subset of 30 transcriptomes that showed the highest relative completeness within each taxon for each of 30 *Oenothera* species and subspecies were chosen for phylogenetic analysis following the BUSCO-based cutoff of <40% of missing and fragmented genes in [82] (S2 Table). For every Trinity “gene” (i.e. unigene) in each of these assemblies, we retained the longest isoform for downstream analysis. Protein-coding genes were predicted from each single-isoform assembly using Transdecoder and validated by searching them against the Swiss-Prot and Pfam databases as noted above. Next, we used the combined set of predicted proteins to infer orthogroups using OrthoFinder v2.4.0 [110,111]. Orthofinder was run with DIAMOND v.0.9.14 [112] for local protein alignment, MAFFT v7.471 [113,114] to generate multiple sequence alignments, and IQ-TREE v.2.0.3 [115,116] for gene tree inference.

To build a phylogeny for all 30 taxa, we used a subset of 1,017 orthogroups, chosen by OrthoFinder, and consisting entirely of single- copy- genes. These orthogroups were then concatenated and aligned across species with MAFFT v7.471 [113,114]. After determining the best-fitting protein substitution model for our data under the BIC criterion with ModelFinder [117]. We generated two phylogenetic hypotheses for *Oenothera* using two distinct methods. The first analysis was conducted based on a concatenated approach using IQ-TREE [115], while the second inferred phylogenetic relationships followed a quartet-based species tree approach using ASTRAL-MP v5.15.5 [118,119]. For the IQ-TREE tree we performed 1,000 ultrafast bootstrap replicates and used nearest neighbor interchange for bootstrap tree optimization [120]. For the ASTRAL analysis, individual genes were inferred using IQ-TREE as noted above. Each of the 1,017 gene trees were input to ASTRAL for species tree inference, which was run using default settings. On the resulting species tree, the internal branch lengths were presented in coalescent units. The branch support values signify a local posterior probability based on the quartet frequencies. The maximum likelihood phylogenies were rooted using *O*. *capillifolia* ssp *berlandieri* as outgroup (S2 Fig). The concatenation and coalescence approach resulted in quite congruent species trees, with good support at the basal nodes. Most of the species-level differences occur within subsection *Oenothera*, where neither tree has particularly good branch support values. Since the two different phylogenies were largely congruent, we employed the concatenation-based tree, which overall showed good node support, for further comparative analysis.

### Comparative analysis

#### Gene family evolution

The output of OrthoFinder was parsed to identify gene families. The species tree based on the concatenated alignments of single-copy orthologs was time-calibrated using the r8s v1.8.1 program [121] with the penalized likelihood method [122]. To provide node age constraints necessary for the time calibration, previously inferred [49] point estimates of divergence time between sister species from four different subsections were used. To assess gene family evolution of the 30 *Oenothera* species and subspecies, we used only the gene families with more than three gene copies per family (23,526) and the species ultrametric tree as inputs to the CAFE v4.2.1106 open access program (Computational Analysis of gene Family Evolution; [121]). The CAFE program uses a stochastic approach to estimate the birth-death (λ) parameter along the provided tree and gene family counts. Lambda describes the probability that any gene will be gained or lost at each node and terminal branch [123], accounting for gene family expansions, contractions. The CAFE software was run using the mode in which the net gain and loss rate are estimated as a single parameter (λ) for each gene family over the whole phylogeny. A strength of the program is that it can detect rapid evolving families, which depict fast rate of genomic turnover (gains and losses per gene per million years). The program estimates the *P* values of gene families in the extant species that are below the threshold. Thus, branches with low *P* values can be regarded as corresponding to gene family expansions and contractions with accelerated evolution rates. On the other hand, a limitation of the program is that it can overestimate gene loss from transcriptome assessment, especially when suboptimal completeness. Hence, we selected the most complete assemblies per species to analyze gene family evolution and employed the longest isoform per gene to avoid redundancies. To ensure transcriptomes were being compared in an equivalent way, all plants were grown in identical conditions and transcriptomes were compared from the same tissue and developmental stage as described above. For the entire analysis, the CAFE overall p-value threshold was kept at its default value (0.01).

#### Defensive gene families and phenolic evolution

To examine the molecular basis for the evolution of secondary metabolism and plant defenses in PTH and sexual plants, we retrieved gene ontology (GO) annotations to search for defense-related genes including those related to eight major phenolic enzymes; PAL, C4H, 4CL, CHS, CHI, F3´H, F3H and FLS. To investigate the potential functions of the Orthofinder results, we assigned defense-related GO terms to each orthogroup if at least one of the proteins in that orthogroup was annotated with the term. We parsed the GO annotations and the orthogroups database to identify putative defense-related proteins encompassed in specific functional categories, and then grouped them under *umbrella* categories according to GO “biological processes” associated with plant defense. To examine the evolution of phenolic metabolism, we tracked phenolic-related genes of each of the 30 taxa. The count and proportion of *defensive* orthogroups and genes corresponding to specific gene ontology categories were then calculated. To investigate whether the observed gene family evolution is associated with functional diversity of chemical plant defense, we retrieved and summarized gene families associated with phenolic biosynthesis for which the rate of gain/loss was significantly different (rapid evolution; *p*-value threshold = 0.01) among taxa.

The protocols employed for phylogenetic and comparative analyses are deposited at dx.doi.org/10.17504/protocols.io.bwnzpdf6.

## Results

### de novo transcriptome data and assembly

Based on Illumina short-read sequencing, 63 transcriptomes from 29 *Oenothera* species (32 taxa including subspecies) were generated [80]. On average, 2.5 billion nucleotides per sample were sequenced [49,80]. After quality filtering, we obtained 30.4 million reads per individual on average. For all sequenced individuals, we produced *de novo* assemblies of mRNA transcripts. Individuals had on average 36,909 assembled transcripts of at least 300 bp in length, and 25.4 Mb per-individual average total length of assembled sequence. Predicted proteins from TransDecoder ranged from 13,291 to 28,379. *Oenothera elata* ssp. *hirsutissima* had the largest number of transcripts (49,655) and *O*. *elata* ssp. *hookeri* had the largest assembly (35M). *Oenothera villosa* ssp. *villosa*, had the lowest number of transcripts (22,547) and assembly length (14 million). (S2 Table). Our 63 *Oenothera* transcriptomes represented 59% of the complete single copy orthologs (BUSCOs) on average, across 29 species PLAZA scores accounted for 86% of conserved gene sets on average, indicating good completeness (S2 Table).

### Annotation and gene ontology (GO) analysis

To provide comprehensive annotation of the 63 transcriptomes, we conducted sequence homology searches of the longest isoform using BLAST tools and retained only protein-coding transcripts for downstream analysis. On average, 84% of sequences matched the annotation databases (blastx hits), ranging from 13,752 (61%) annotated genes of *O*. *villosa* ssp. *villosa*, to 38,200 (98%) of *O*. *filiformis* (S3 Table). From blasted genes (transcripts with positive hits), 71% of sequences matched with Pfam’s database and more than 80% were associated with GO terms. We identify a wide range of GO terms in each assembly, indicating that three functional categories; cell component, molecular function and biological process, were well represented (S3 Fig).

### Comparative transcriptomic analyses

Protein coding genes from 30 transcriptomes were used to construct orthogroups (gene families). There were 670,846 genes out of 681,746 (94.8% of total) organized in orthogroups or protein families (S4 Table). The mean and median of gene family size is 25.3 and 36 proteins (G50 = 36), respectively. We found 7,760 families present in all species, whereas 1,071 consisted entirely of single copy-genes.

**Fig 1:**
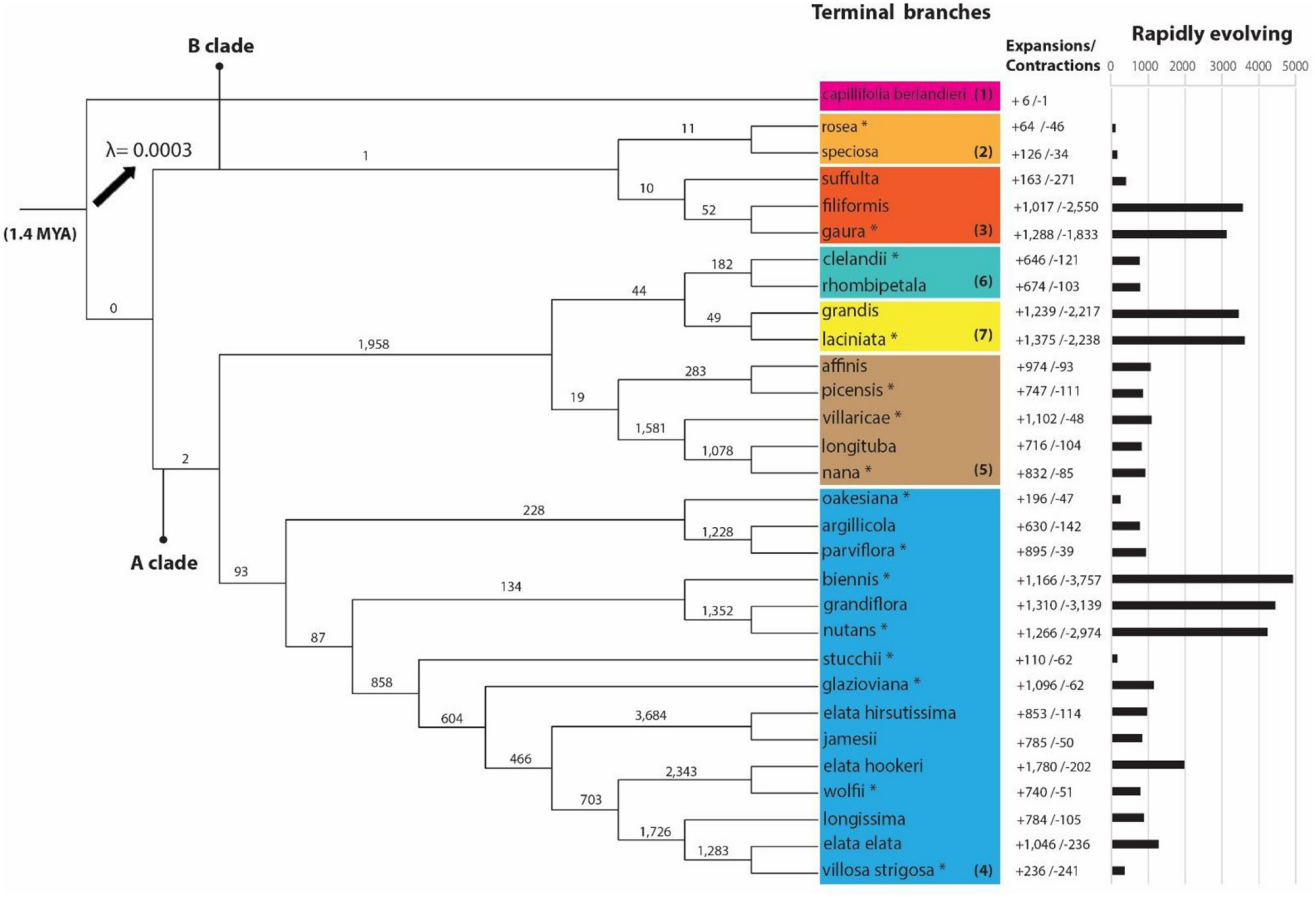
Phylogenetic time tree constructed from concatenated alignment of 1,017 orthogroups consisting entirely of single-copy genes from 30 *Oenothera* taxa with *Oenothera capillifolia* ssp. *berlandieri* from subsection *Calylophus* (1) as outgroup. Seven sections/subsections are depicted. Two major clades A and B include: section *Hartmannia* (2), subsection *Gaura* (3), subsection *Oenothera* (4), subsection *Munzia* (5), subsection *Candela* (6) and subsection *Raimannia* (7). Asterisks depict PTH species. The number of significant gene family expansions (+), contractions (-) and rapidly evolving gene families resulted from CAFE analysis are shown on terminal branches. Also, the number of rapidly evolving families are depicted above internal branches. The rate of gene gain and lost (lambda) for the whole tree was 0.0003.

The species phylogeny included seven sections and subsections of *Oenothera* showing two major clades. Clade A comprises all subsections of section *Oenothera*, (i. e., *Oenothera*, *Munzia*, *Candela* and *Raimannia*), whereas clade B includes section *Hartmannia* and subsection *Gaura*. *Oenothera capillifolia* ssp. *berlandieri* (section *Calylophus*) rooted the tree at about 1.4 MYA. These data tentatively suggest that Clade A diverged ~ 0.95 MYA from clade B. The rate of gene gain and loss (λ) estimated from the CAFE analysis was 0.0003 genes over time, for the entire tree (Fig 1). Some of the fastest gene family evolution occurred within subsection *Oenothera*. For example, the internal branch with the largest number of rapidly evolving gene families corresponds to the most recent common ancestor of *O*. *elata* ssp. *hirsutissima* and *O*. *jamesii*. The terminal branch with the most rapidly evolving gene families and largest significant gene family contractions is the one leading to *O*. *biennis*; this subclade also included the only two other species that showed more than 4,000 rapidly evolving gene families (*O*. *grandiflora* and *O*. *nutans*). *Oenothera elata* ssp. *hookeri* showed the highest number of significant gene family expansions, whereas *O*. *capillifolia* ssp. *berlandieri* from section *Calylophus* had the least significant expansions and contractions (Fig 1).

### Defense-related gene families and phenolics evolution

We identify two major biological processes involved in plant defense and secondary metabolism: 1) regulation of defense response, including response to fungi, bacteria, viruses, nematodes, and insects, and 2) regulation of secondary metabolites and plant hormones, which includes, the phenolic biosynthetic process and hormone-based metabolism. We found 61 GOs related to plant defense and secondary metabolism, from which 74% corresponded to regulation of defense response, and the other 26% belonged to regulation of secondary metabolites and plant hormones (Table 1). Response to fungi and oomyecetes involved the highest number of GO categories (12). Response to insect herbivores and wounding involved 7 GO categories associated with 386 non-exclusive orthogroups (i.e., orthogroups including proteins annotated in diverse GO categories). The shikimate and phenolic biosynthesis showed 8 GO categories, the highest number of categories out of the regulation of secondary metabolism and plant hormones processes. The hormone-based responses, which include the jasmonic acid and salicylic acid metabolic pathways, showed the highest number of non-exclusive orthogroups (Table 1). A full list of defense-related GO categories and orthogroups is provided in S5 Table.

**Table 1:** Functional categories from 30 *Oenothera* taxa of orthogroups related to defense and secondary metabolism with gene ontology (GO) annotations. For each GO biological process, we provide the sum of annotations related to gene ontology categories, a representative accession number of each category and the number of orthogroups associated with annotations of specific categories.

Biological process	# GO category	GO accession number	# Orthogroups
***Regulation of defense response***			
Immune response	5	0045087	240
Defense response	5	0006952	1125
Response to fungus and oomycetes	12	0050832	1028
Response to bacteria	9	0042742	1062
Response to virus	4	0051607	110
Response to nematode and other organism	3	0002215	20
Defense response to insect/ herbivore and wounding	7	0009625	386
***Regulation of secondary metabolites and plant hormones***			
Secondary metabolism	1	0019748	16
Shikimate and phenolic biosynthetic process	8	0009813	201
Hormone-based response (ethylene, jasmonic and salicylic acid metabolism)	7	0009753	724

Note. When a biological process included more than one GO category (gene ontology name), a representative GO accession number was provided.

**Fig 2:**
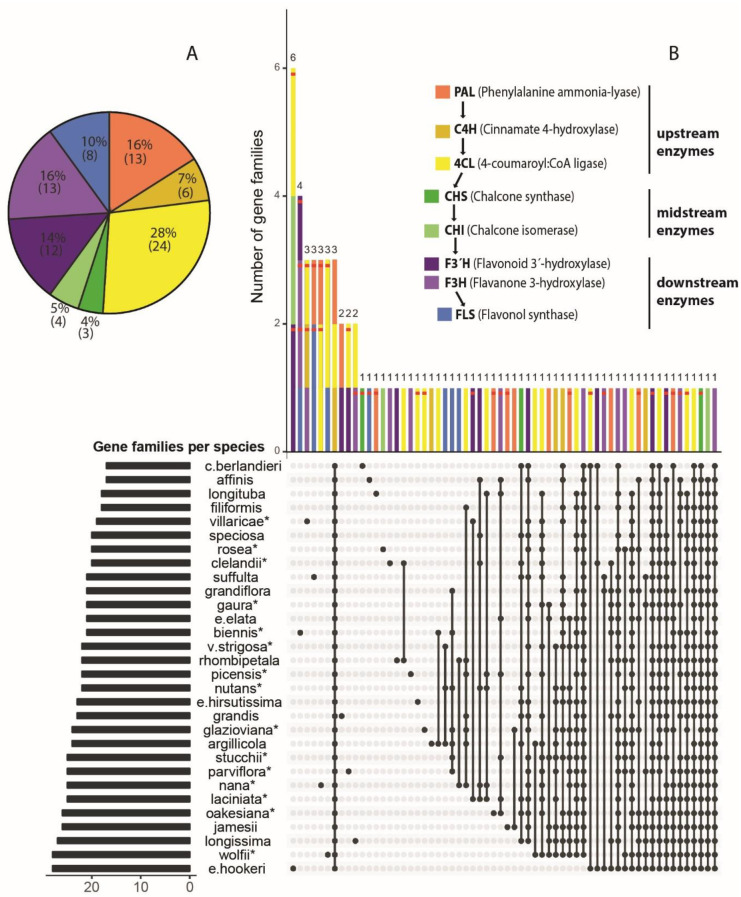
Distribution of phenolic-related genes in 30 *Oenothera* taxa. (A) The relative proportion of 83 gene families related to major enzymes involved in the synthesis of phenolic compounds. (B) shows intersections of major phenolic up- mid- and downstream enzyme related-genes from *Oenothera* transcriptomes. In the upper panel each color bar corresponds to a single or a set of gene families matching to specific pattern of species intersection. Red-striped bars indicate 33 rapidly evolved gene families based on the CAFE analysis. Filled circles in the bottom panel indicate the presence of phenolic-related genes per taxa. Connected circles indicate shared gene families among taxa. Lower left panel indicate the number of gene families corresponding to each filled-circle pattern.

To analyze the evolution of phenolic metabolism across 30 *Oenothera* taxa, we quantified gene families with annotations for major phenolic enzymes, PAL, 4CL, C4H, CHS, CHI, F3´H, F3H and FLS, and accounted for those exhibiting rapid evolution. In total, we identified 83 phenolic-related gene families, of which more than half (43) correspond to the “core” phenylpropanoid pathway (PAL, 4CL and C4H). Chalcone enzymes (CHS and CHI) comprised ~10% of phenolic proteins and downstream enzymes involved in the synthesis of flavonoids, coumarins and tannins, among others, represented ca. 40%. The 4CL enzyme showed the highest proportion of phenolic proteins (28%) and the CHS the least proportion (3%) (Fig 2A). Phenolic gene families per species ranged from 28 (*Oenothera elata* ssp. *hookeri and O*. *wolfii*) to 17 (*O*. *capillifolia* ssp. *berlandieri and O*. *affinis*). Subsection *Oenothera* and *Munzia* comprised species with the largest amount of shared and species-specific phenolic gene families, consistent with their monophyletic relationship within *Oenothera* clade A. We identified 40 species-specific gene families distributed across 21 taxa of the seven subclades. *Oenothera elata* ssp. *hookeri* comprised 6 species-specific gene families, followed by *O*. *biennis* with 4, and by *O*. *villaricae*, *O*. *suffulta*, *O*. *nana* and *O wolfii* with 3 families by each species. *Oenothera wolfii*, *O*. *jamesii* and *O*. *oakesiana* showed the highest number of intersections with other species (23), whereas *Oenothera capillifolia* ssp. *berlandieri* and *O*. *affinis* showed the least number of shared gene families (14). About 70% of phenolic families occurred in < 10 species, whereas 20% of the families occurred in ≥ 20 species. Three gene families of the “core” phenylpropanoid occurred in all taxa, and two families of the flavonoid branch occurred in all but one taxon. However, except for the three families occurring in the 30 taxa, every gene family shared by at least two taxa showed a unique pattern of intersection, never involving the same species (Fig 2B).

About 40% of phenolic gene families rapidly evolved according to CAFE analysis. Both PAL and 4CL enzymes accounted for most of the significant phenolic evolution, with 8 and 9 gene families, respectively (Table 2). C4H and FLS involved the least number of rapidly evolving families, with one and two, respectively. CHI did not show significant evolution when measured as the rate of gene family expansions or contractions.

**Table 2:** Evolution of phenolic gene families across 30 *Oenothera* taxa as determined by CAFE analysis of seven enzymes; PAL (phenylalanine ammonia-lyase), 4CL (4-coumaroyl: CoA ligase), C4H (Cinnamate 4-hydroxylase), CHS (Chalcone synthase), F3´H (Flavonoid 3´-hydroxylase), F3H (Flavanone 3-hydroxylase) and FLS (Flavonol synthase). We show the total number of gene families that are rapidly evolving (rapid evolution; *p*-value threshold = 0.01), the number of gene families and genes that have experienced expansions contractions. The percentage per each of the seven phenolic enzymes is shown in parentheses.

Enzyme class	RAPIDLY EVOLVING	EXPANSIONS		CONTRACTIONS	
	Gene family	Gene family	Gene gain	Gene family	Gene loss
**PAL**	8 (24)	7 (22)	20 (16)	3 (19)	4 (10)
**4CL**	9 (28)	9 (28)	34 (26)	8 (50)	23 (58)
**C4H**	1 (3)	1 (3)	1 (1)	1 (6)	1 (2)
**CHS**	2 (6)	2 (6)	6 (5)	0	0
**F3´H**	5 (15)	5 (16)	23 (18)	2 (13)	9 (23)
**F3H**	6 (18)	6 (18)	27 (21)	1 (6)	2 (5)
**FLS**	2 (6)	2 (7)	17 (13)	1 (6)	1 (2)

It is interesting to note that most species-specific gene families experienced rapid evolution. Of the 21 species that showed rapid evolution in species-specific gene families, six species involved ≥ 2 families. *Oenothera biennis* is the only species that showed rapid evolution in almost all its species-specific gene families (three out of four), which are involved in the synthesis of flavonoids (F3´H and F3H). Chalcone synthesis showed contrasting patterns: whereas CHI families showed no rapid evolution, CHS was the only enzyme with a significantly evolved family present in almost all species (28). Only one phenolic family, the last major branching enzyme FLS, showed rapid evolution (Fig 2B).

We also examined gene gain and loss resulting from expansions and contractions of phenolic families. Gene family expansion involved almost all phenolic families (32 out 33), whereas gene family contraction involved about half of families (16). During *Oenothera* diversification, phenolic families gained 128 genes of which 28% corresponded to 4CL. This enzyme also comprised the largest gene loss (58%) within the genus. Branching enzymes F3H and F3´H comprised the second highest number of gained genes with 27 and 18 genes, respectively. Upstream C4H and midstream CHS showed the least gene gain, with one and five genes, respectively. Branching enzymes F3H and FLS, along with C4H loss the least number of genes (≤ 1). CHS families are the only ones exhibiting gene gain and no losses.

### Phenolic evolution and the genetic system

**Fig 3:**
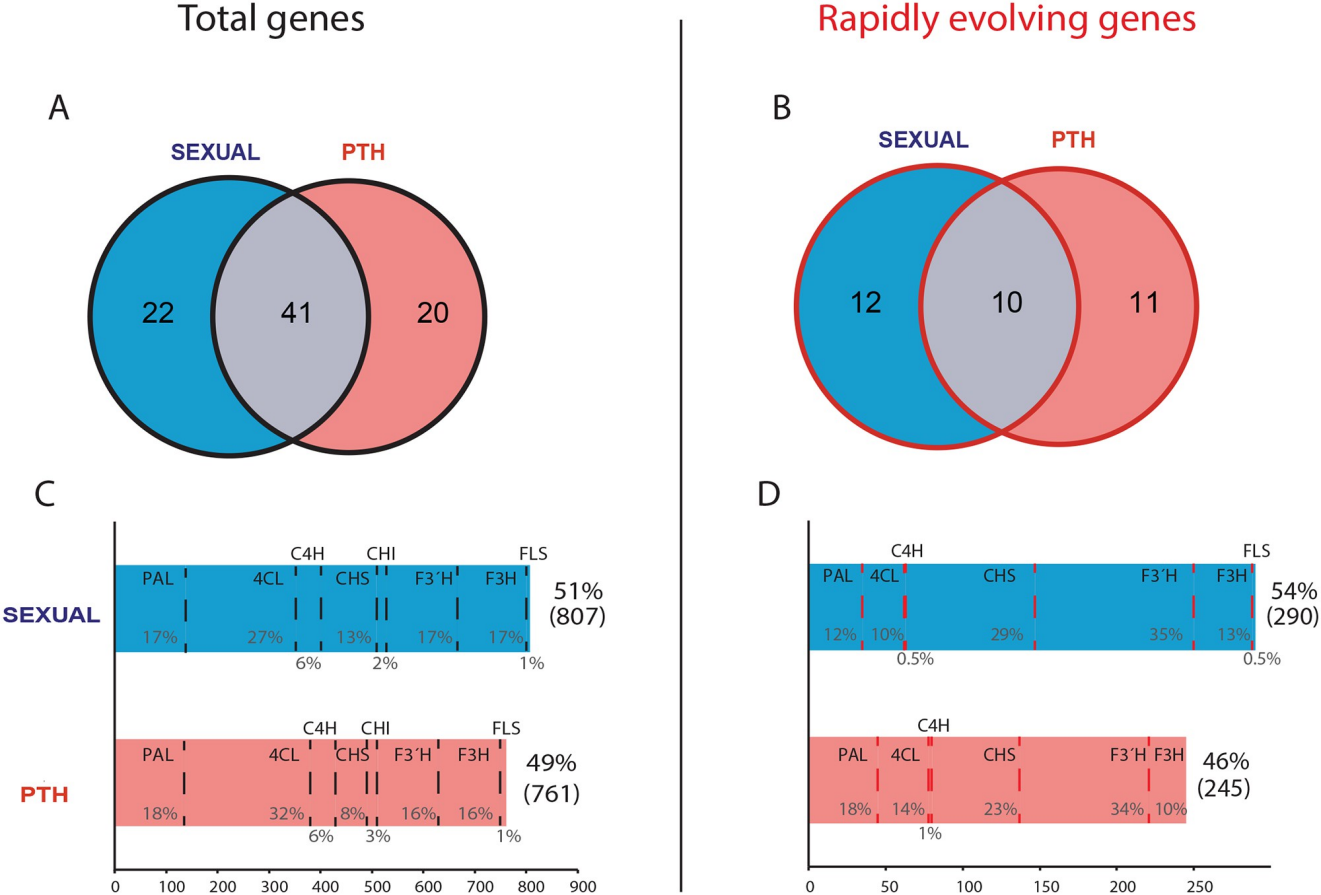
Distribution of phenolic-related genes of 15 sexual and 15 PTH *Oenothera* taxa. Venn diagrams show the intersection between sexual and PTH plants including (A) 83 phenolic-related gene families and (B) 33 rapidly evolving families based on CAFE analysis. Bar charts depict the summary of transcripts related to phenolic enzymes stemming from (C) total gene family count and (D) rapidly evolving gene families.

We analyzed the distribution of phenolic-related genes between 15 sexual and 15 PTH taxa. Of the 83 gene families with phenolic enzyme annotations, almost half are shared between sexual and PTH plants, while ~25% are specific to sexual and the other 25% to PTH taxa (Fig 3A). On the other hand, of the 33 rapidly evolving phenolic families, about 33% are shared between sexual and PTH species, and 66% are specific to either sexual or PTH species (Fig 3B). We successfully identified 1,568 proteins with annotations of major phenolic enzymes from the total phenolic family count, of which 51% correspond to sexual taxa and the rest to PTH taxa (see S6 Table for species count of phenolic proteins). The relative proportion of enzymes between sexual and PTH plants is highly consistent. 4CL accounted for most of the proteins (27–32%), followed by PAL (~ 18%), F3´H and F3H (~ 17%), CHS (< 15%) C4H (~ 6%) CHI (~ 2%) and FLS (1%) (Fig 3C). Rapidly evolving families comprised 535 proteins, from which 54% correspond to sexual plants and the rest to PTH. Although the amount of rapidly evolving proteins was mostly consistent between sexual and PTH taxa, proteins of specific phenolic enzymes differed in their rate of evolution (expansion/contraction) between the two reproductive types. Whereas 4CL proteins were the most widely distributed in the total gene families, when non-rapidly evolving families were excluded, CHS and F3´H became the dominant enzymes, accounting for more than a half of the phenolic proteins in both sexual and PTH taxa. It is important to note that CHS is the enzyme that involves the least number of phenolic gene families (3), but at the same time comprised between 29% and 23% of all proteins that evolve significantly, in sexual and PTH taxa, respectively. Although the overall proportion of phenolic-related proteins was higher in sexual plants, PTH taxa showed a higher number of genes related to upstream enzymes involving 429 and 80 proteins in both the total count and rapidly evolving gene families, respectively (Fig 3C and 3D).

## Discussion

Our study analyzes genomic evolution in the genus *Oenothera*, and address gene family diversification with special focus on phenolic metabolism. Two results from our analysis are particularly important for answering our research questions. First, we found a large number of gene families exhibiting rapid evolution, yet there was large heterogeneity in gene family evolution across the genus, with section *Oenothera* exhibiting most of the largest expansions and contractions, and significant evolutionary changes. Second, when we focused on molecular evolution of phenolic enzymes, which are involved in secondary metabolism including defense, we observed that ca. 40% of the phenolic gene families exhibited rapid evolution during diversification, involving genes encoding for up-, mid-, and downstream enzymes. We discuss the importance of these results for the macroevolution of *Oenothera*, the evolution of phenolic metabolism and plant defense.

### Transcriptomic resource

We described 63 *de novo* assembled leaf transcriptomes from 29 *Oenothera* species, encompassing the natural variation in the genetic system (PTH vs sexual), geographic distribution, reproductive and defensive chemical traits, among others, across seven subclades. Our new assemblies increased the number of annotated genes by 20% compared to a previous assessment [49], providing a wide view of functional diversity in protein-coding genes. BUSCO gene sets indicate fairly complete transcriptomes given that a single tissue was sampled, yet these varied over 2-fold among taxa. The use of the PLAZA comparative resource showed higher values of relative completeness and less variation among species. Altogether these scores indicate the quality of RNA samples, sequencing, assembly, and gene prediction We believe this transcriptomic resource will be useful in addressing many problems in plant biology, including the systematics of *Oenothera*, the functional roles and divergence of proteins, the role of gene family diversification in molecular, phenotypic and species diversification, the genomic consequences of sexual reproduction and hybridization, and the evolution of plant defence.

### Major patterns of gene family evolution

There was large variation in gene family evolution across *Oenothera*. We found three major patterns: 1) across the genus, gene expansions were more common than contractions; 2) the most rapidly evolving gene families accounted for larger contractions than expansions; and 3) subsection *Oenothera* comprised species with the largest rapid evolution. Leebens-Mack et al. [82] addressed gene family evolution in the 1kp project, across green plants (Viridiplantae), including representative *Oenothera* species and sister clades, but did not detect significant expansion/contractions on major gene families within Rosids. Wide scale examination of gene family evolution at large phylogenetic scales is likely to overlook finer-scale evolutionary processes. Our study is the first in addressing gene family evolution not only in *Oenothera* but also within the Onagraceae, documenting the genomic variability of 32 different taxa (29 species) and the evolutionary dynamics to which protein families have been subject to during the last 1.4 MYA.

The large variation in the number of gene family expansions and contractions that we observed may be influenced by different factors, such as gene duplication, *de novo* gene creation, gene loss, and changes in environmental conditions [124,125]. Studies have shown a key role of gene duplication for the evolution of new genes and gene families [126,127], leading to increased protein yield and neofunctionalization during divergence [128]. In *Oenothera*, hybridization plays an important role in speciation, simultaneously causing the fixation of diverged alleles in the heterozygous state when hybridization is accompanied by functionally asexual PTH reproduction [13,50]. Heterozygous PTH genotypes involve heterogeneous gene duplications (combining divergent alleles of a single locus), which creates new material for long-term genetic innovation, contributing to gene family proliferation. Although in our study the number of taxa exhibiting larger gene family expansions was similar between sexual and PTH species, notably, the functionally asexual species *O*. *biennis* showed disproportionate gene family contractions accounting for the highest rapid evolution across the genus. On the other hand, sexual *O*. *elata* ssp. *hookerii* accounted for the largest gene gains. These results showing that rates of gene family evolution are overlapping between PTH and sexual taxa, even though the mechanisms causing gene gains and losses might be distinct. Nonetheless, gene loss and contractions should be taken with caution due to limitations of transcriptomic analyses.

*Oenothera* subclades showed contrasting divergence of gene families. Subsections *Gaura*, *Raimannia*, and *Oenothera* exhibited the greatest gene expansions (>3,000 gene families change over time in at least one species per clade), whereas subsection *Calylophus* and section *Hartmannia* exhibited the slowest gene expansion (<200 gene families change over time by species). Subsection *Oenothera* is the largest group included in this study; it also has the most rapid evolution across the genus. This group of 11 species native to North America has expanded its range within the last 400 years to other continents, including Europe [1,32,129,130]. Rapid climate change following deglaciation also led to rapid range expansions and new environmental conditions for these species [45,57]. All these factors may have imposed both strong and varying selection, coupled with prominent neutral evolutionary processes through drift and gene flow, which might have contributed to rapid gene family evolution.

### Genomic evolution of plant defense and phenolic metabolism

We assessed the evolution of genes implicated in plant defense, secondary metabolism and more specifically phenolic biosynthesis. Response to pathogens comprised the most annotated proteins, followed by response to wounding, insect herbivores and other invertebrates. A review of pathogenesis ontology showed a large set of GO terms corresponding with our annotation, implicated in response to oomycetes [131]. The regulation of secondary metabolism of defense-related compounds (phenolic compounds and phytohormones) was less extensive, comprising about a third of the GO terms of the regulation of plant defense, in agreement with annotations of secondary metabolism in species of the same biosynthetic pathway [132]. Most annotated proteins with these GO categories are ubiquitous in plants, depicting common functional complexes resulting from species interactions and life history. This set of GO terms provides a solid base to further compare and contrast the molecular underpinnings of plant defense and secondary metabolism.

Phenolic metabolism involved 1,568 common genes arranged into 83 gene families that vary widely across the genus *Oenothera*. The genes encoding for the eight major phenolic enzymes occurred in all clades except Section *Calylophus* (which lacked CHI in our assembly), although they were differentially spread among taxa. In our study, upstream enzymes PAL, 4CL and C4H of the core phenylpropanoid complex comprised most of the phenolic proteins (51%) occurring in *Oenothera*, followed by downstream enzymes F3´H, F3H and FLS implicated in branching synthesis of final compounds, which covered 40% of phenolic families, and by midstream CHS and CHI, which channelize precursors into branches and had the smallest fraction of phenolic families covering about 10% of proteins. Each of these up-, mid-, and downstream enzymatic complexes also show an uneven distribution among species. For instance, species such as *O*. *filiformis* and *O*. *jamesii* had the largest PAL- and 4CL-related genes, with 17 and 27 proteins, respectively, but a relatively low count of mid- and downstream proteins. Our results show that orthologous genes of phenolic metabolism are well conserved across *Oenothera* and are consistent with comparative studies documenting the uneven size of multigene phenolic families across angiosperms [133–140]. Differences in the presence and copy number of phenolic genes across species documented in our study, gives clues as to the evolutionary dynamics of the genus and the evolution of phenolic metabolism and their enzymatic complexes.

All taxa experienced rapid evolution of phenolic proteins, involving 33 gene families, with an average of six families per species (Table 2). Overall, rapidly evolving phenolic families experienced greater expansions than contractions, gaining about 2-fold more genes than they lost. Upstream enzymes PAL and 4CL of the core phenylpropanoid complex comprised most of the rapidly evolving gene families, both enzymes contained the largest expansion and the latter exhibited the largest contraction (40% of 4CL genes were lost) during diversification. Our results are consistent with the expectation that upstream enzymes of greatest control over flux in metabolic pathways experience disproportionate evolutionary changes [141–144]. In addition, consistent with expectation of rapid evolution of downstream complexes as subject to reduce selective constraint [145,146], we found that branching enzymes F3´H, F3H and FLS accounted for more than half of genes gained [67], involving about 40% of gene families. Notably, chalcone genes that accounted for the smallest gene family set, were the only ones experiencing expansions and no contractions.

Variation across phenolic complexes of rapidly evolving genes also showed contrasting patterns among *Oenothera* taxa and clades, ranging from 3 up to 10 families per species. One significantly evolved gene family (Orthogroup ID: OG0002469) of CHS metabolism, occurred in almost all taxa (except for *O*. *biennis* and *O*. *clelandii*), whereas most rapid evolution focused on species-specific families. *Oenothera biennis* and *O*. *elata* ssp. *hookeri* of subsection *Oenothera* comprised most of the rapid evolution of species-specific families. In fact, this subsection comprised species with the largest rapid phenolic evolution, with five taxa *O*. *oakesiana*, *O*. *elata* ssp. *hirsutissima*, *O*. *elata* ssp. *hookeri*, *O*. *jamesii*, *O*. *wolfii* and *O*. *longissima* having ≥ 9 rapidly evolving phenolic families. These divergent patterns can be associated with changes in ecological factors such as climate and the biotic environment. Plant phenolics play a key role as defense mechanism against herbivores [147–150]. In *Oenothera*, it has been shown that the level of phenolic-based defenses increased in cold climates at higher latitudes [59], where most of the rapidly evolving species occurred (e.g., *O*. *oakesiana* and *O*. *wolfii* grow in regions of mean annual temperature below 11°C). This suggests that adaptive response to the abiotic and biotic environment may have influenced the proliferation of phenolic families during *Oenothera* diversification. These adaptive responses are likely to influence fine-tuning changes along the biosynthetic machinery. For instance, *O*. *biennis* which produces a wide range of flavonoids and hydrolysable tannins unique in the plant kingdom and subject to ongoing natural selection [52,53], experienced the largest evolutionary changes of exclusive gene families involved in the synthesis of flavonoids (F3´H and F3H).

Although empirical evidence across *Oenothera* has confirmed classic predictions of decreased levels of defense with reduced sexual reproduction [44] (albeit increased diversity of flavonoid metabolites [48], our results showed less clear differences between functionally asexual PTH and sexual plants. We found that although sexual and PTH plants are well differentiated having about 30% (total) and 50% (rapidly evolving) specific gene families each, both groups had almost the same number of phenolic proteins. However, we found differences in the relative proportion of proteins encoding different enzymes among sexual and PTH plants. For instance, PTH plants had ~20% more rapidly evolving genes of upstream enzymes than sexual plants. In contrast, sexual plants had ~30% more rapidly evolving mid- and downstream genes than asexual plants. This suggests that although PTH and sexual plants share a common gene catalog, the mating system may contribute to molecular changes in specific phenolic complexes.

## Conclusion

The transcriptomic resource developed here provides a rich and powerful tool for the comparative study of plant biology, especially as it relates to systematics, the study of secondary metabolism, plant sexual reproduction and genomic evolution more generally. We analyzed the molecular evolution of secondary metabolism and plant defense. By identifying orthologous genes across taxa combined with phylogenetic and gene family evolution analysis, we revealed a variable number of molecular changes resulting in genomic expansions and contractions o that have also shaped biosynthetic phenolic genes. Integration of pathway-level genomic, transcriptomic data and phylogenetic approaches provides a clearer framework to illustrate differences among species. The molecular resource we present are intended to provide a further step to build a predictive gene framework for understanding the evolutionary forces driving population, community and species-level diversity in the historically significant model organism *Oenothera*.

## Supporting information

S1 Fig Schematic of the general phenylpropanoid, isoflavonoid, flavonoid pathways. (DOCX)

S2 Fig Maximum likelihood species trees inferred from 1,017 orthogroups consisting entirely of single-copy genes from 30 *Oenothera* taxa with *O*. *capillifolia* spp *berlandieri* as outgroup. (DOCX)

S3 Fig Functional diversity across 30 *Oenothera* taxa, according to gene ontology (GO) nomenclature. (DOCX)

S1 Table Taxonomic, sexual system and collection data from 63 *Oenothera* samples used for transcriptomes assembly. (XLSX)

S2 Table Overview of the RNA-seq assembly of 63 *Oenothera* transcriptomes and summarized of BUSCO and PLAZA scores. (XLSX)

S3 Table Annotation stats of blasted annotated genes based on the longest isoform from 63 *Oenothera* individuals. (XLSX)

S4 Table OrthoFinder statistics for orthogroup construction of 30 *Oenothera* taxa. (DOCX)

S5 Table Gene count for each gene ontology category and associated orthogroups identified across 30 *Oenothera* transcriptomes. (XLSX)

S6 Table Count of phenolic proteins in 30 *Oenothera* taxa. (XLSX)
