# Supplementary material for: The evolution of multi-gene families and metabolic pathways in the evening primroses (Oenothera: Onagraceae): A comparative transcriptomics approach
Source: PLoS One. 2022 Jun 24;17(6):e0269307. doi: 10.1371/journal.pone.0269307 (PMC9231714; doi:10.1371/journal.pone.0269307)
Supplement: S1 Fig — (DOCX) [file pone.0269307.s001.docx]

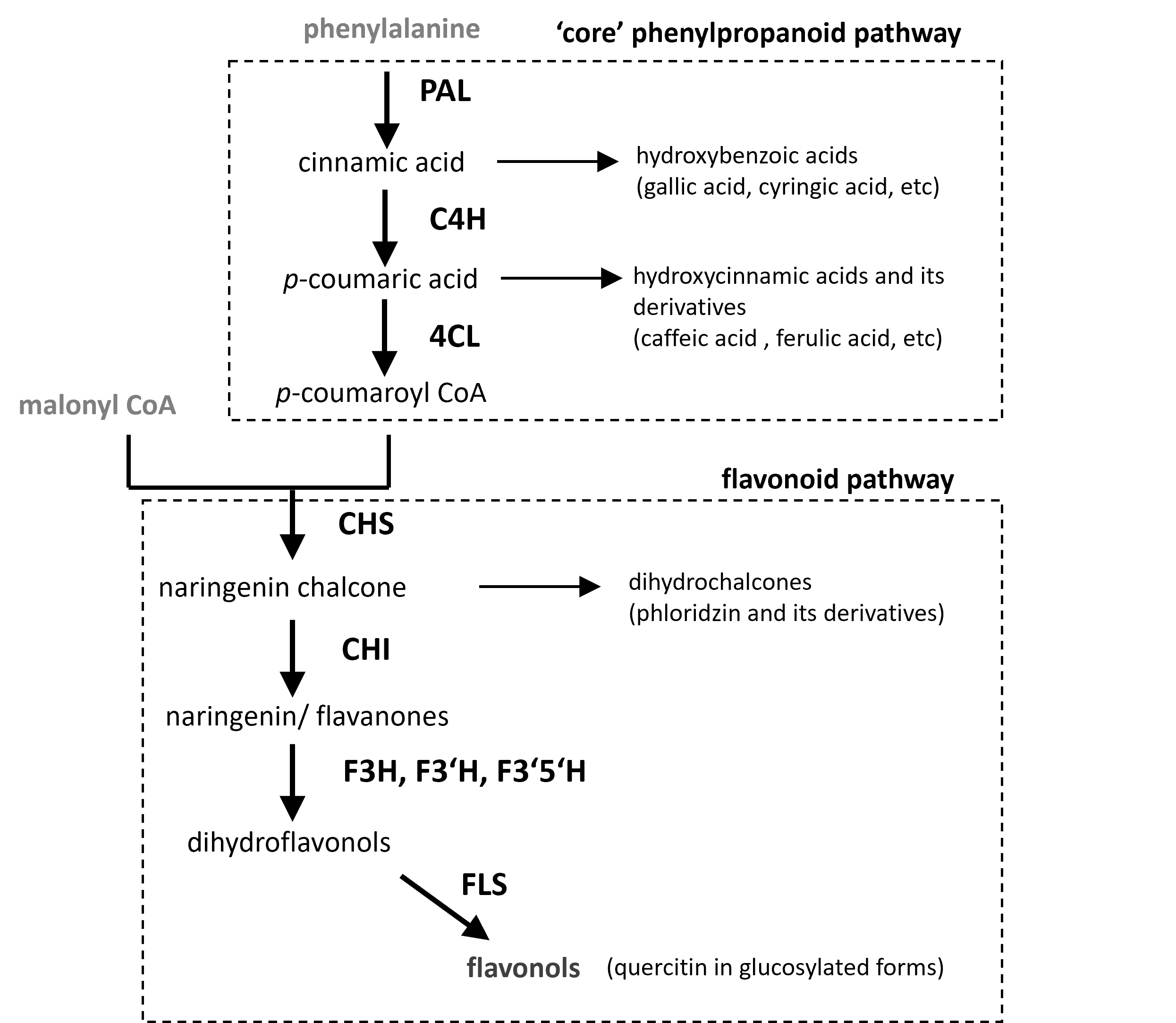


**Figure S1**. Schematic of the general phenylpropanoid, isoflavonoid, flavonoid pathways. Enzymes are indicated in bold, abbreviated as follows C4H; cinnamate 4-hydroxylase, 4CL; 4-coumaroyl:CoA ligase, CHS; chalcone synthase, CHI; chalcone isomerase, F3H; flavanone 3-hydroxylase, F3´H and F3′5′H; flavonoid 3′ and 3′5′ hydroxylase, FLS; flavonol synthase and PAL; phenylalanine ammonialyase. Modified from Winkel-Shirley 1999 and Kalinowska et al. 2014.
