# Supplementary material for: The evolution of multi-gene families and metabolic pathways in the evening primroses (Oenothera: Onagraceae): A comparative transcriptomics approach
Source: PLoS One. 2022 Jun 24;17(6):e0269307. doi: 10.1371/journal.pone.0269307 (PMC9231714; doi:10.1371/journal.pone.0269307)
Supplement: S2 Fig — (DOCX) [file pone.0269307.s002.docx]

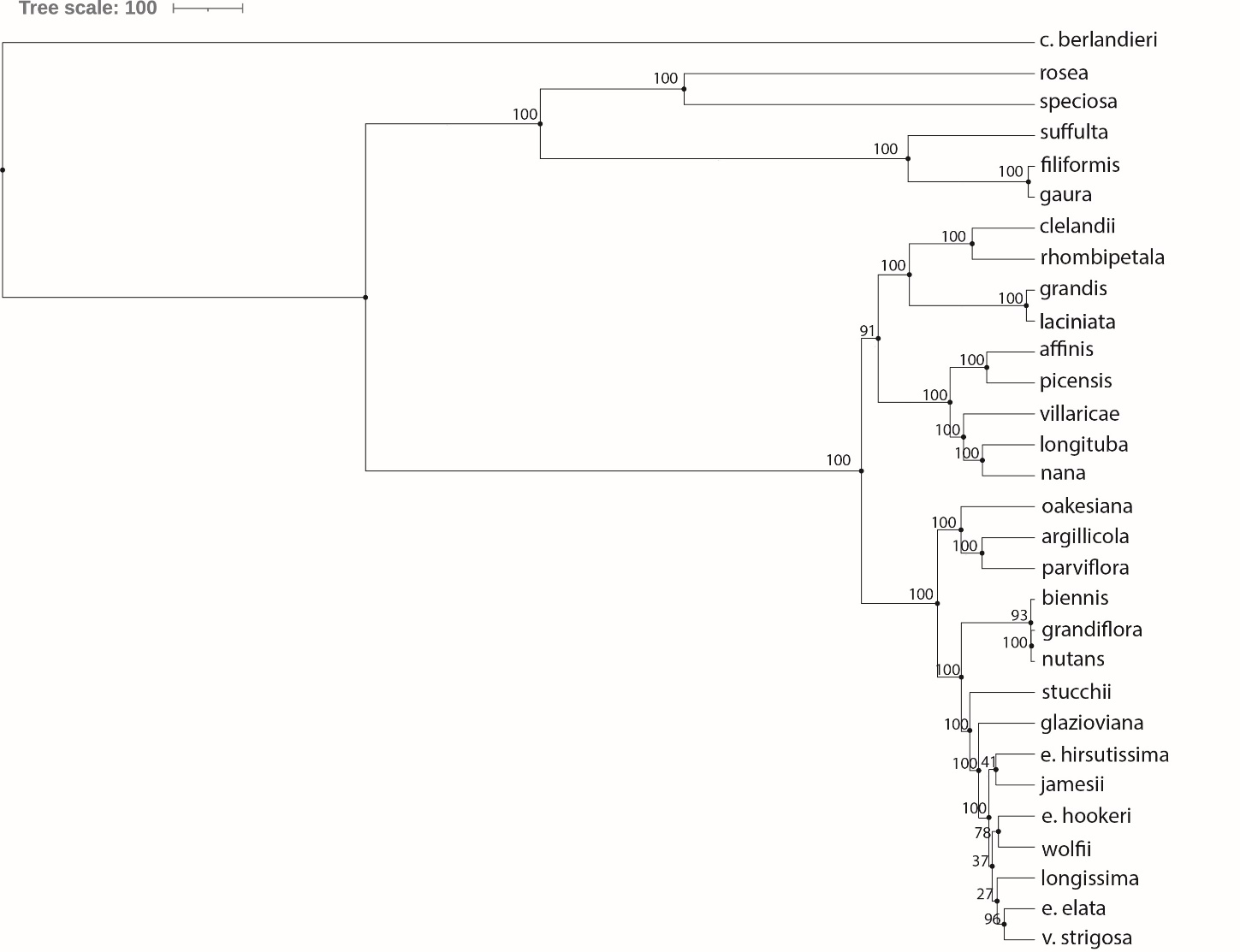


**A**


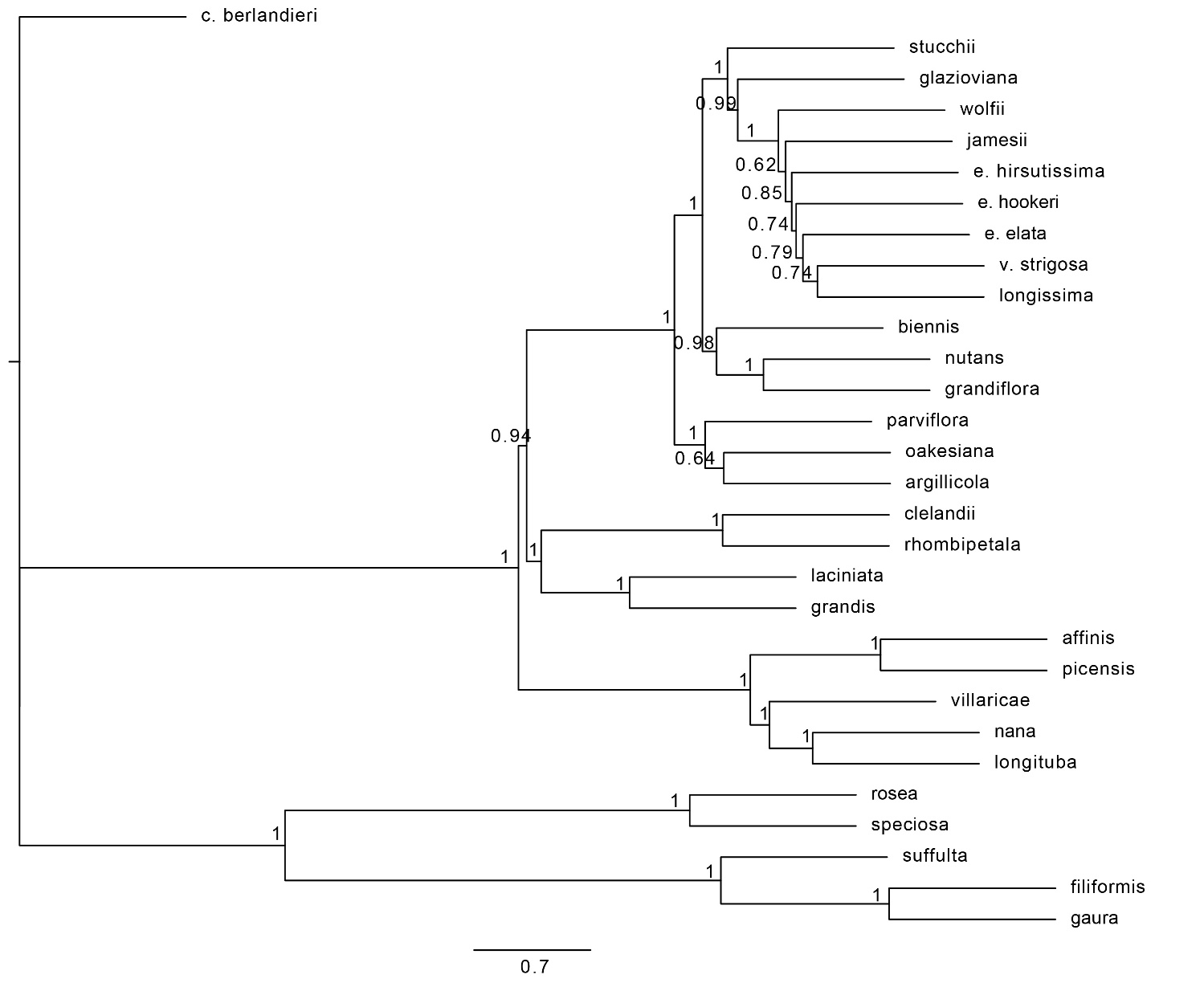


**B**

**Figure S3**. Maximum likelihood species trees inferred from 1,017 orthogroups consisting entirely of single-copy genes from 30 *Oenothera* taxa with *O.* *capillifolia* spp *berlandieri* as outgroup. A) depicts IQ-TREE tree with bootstrap values supporting the tree on top of each node. B) ASTRAL tree with support values (local posterior probability based on the quartet frequencies) on top of each node.
