# Supplementary material for: The evolution of multi-gene families and metabolic pathways in the evening primroses (Oenothera: Onagraceae): A comparative transcriptomics approach
Source: PLoS One. 2022 Jun 24;17(6):e0269307. doi: 10.1371/journal.pone.0269307 (PMC9231714; doi:10.1371/journal.pone.0269307)
Supplement: S3 Fig — (DOCX) [file pone.0269307.s003.docx]

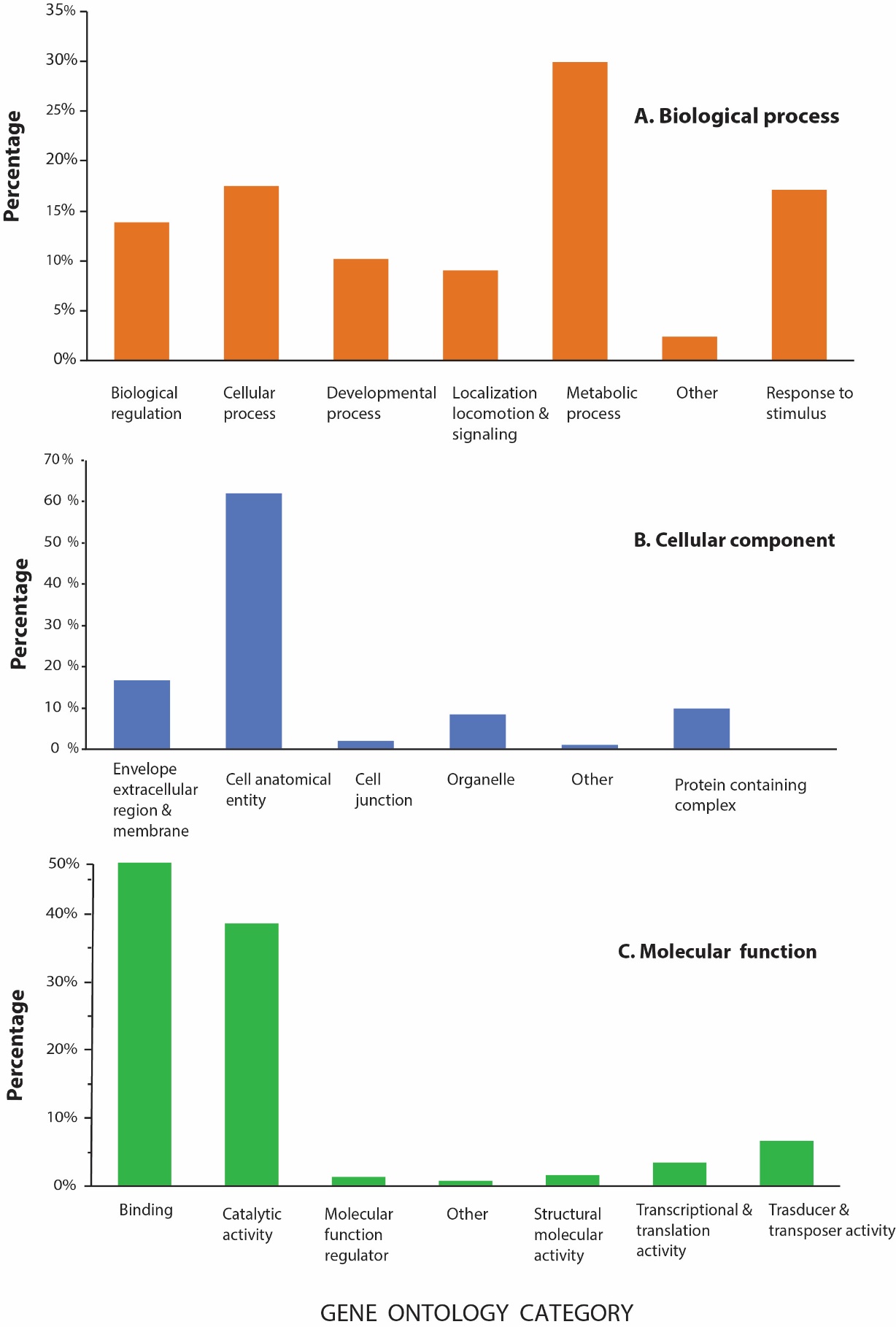


**Figure S3**. Functional diversity across 30 *Oenothera* taxa, according to gene ontology (GO) nomenclature.
