# Supplementary material for: The evolution of multi-gene families and metabolic pathways in the evening primroses (Oenothera: Onagraceae): A comparative transcriptomics approach
Source: PLoS One. 2022 Jun 24;17(6):e0269307. doi: 10.1371/journal.pone.0269307 (PMC9231714; doi:10.1371/journal.pone.0269307)
Supplement: S4 Table — (DOCX) [file pone.0269307.s007.docx]

| **Table S4**. OrthoFinder statistics for orthogroup construction of 30 *Oenothera* taxa. | |
| --- | --- |
| Number of genes | 681,746 |
| Number of genes in orthogroups | 670,843 |
| Number of unassigned genes | 10,903 |
| Percentage of genes in orthogroups | 98.4 |
| Percentage of unassigned genes | 1.6 |
| Number of orthogroups | 26,555 |
| Number of species-specific orthogroups | 162 |
| Number of genes in species-specific orthogroups | 354 |
| Percentage of genes in species-specific orthogroups | 0.1 |
| Mean orthogroup size | 25.3 |
| Median orthogroup size | 25 |
| G50 (assigned genes) | 36 |
| Number of orthogroups with all species present | 7,760 |
| Number of single-copy orthogroups | 1,017 |
